# A Rare Case of Myxedema Coma Presenting as Bradycardia and Hypotension Secondary to Pituitary Apoplexy

**DOI:** 10.7759/cureus.15196

**Published:** 2021-05-23

**Authors:** Sukhdeep Bhogal, Nirav Patel, Kajal Mawa, Vijay Ramu, Timir Paul

**Affiliations:** 1 Internal Medicine, East Tennessee State University, Johnson City, USA; 2 Internal Medicine, Johnston Memorial Hospital, Abingdon, USA; 3 Internal Medicine, Dayanand Medical College and Hospital, Ludhiana, IND; 4 Cardiology, East Tennessee State University, Johnson City, USA

**Keywords:** myxedema coma, pituitary apoplexy, prolonged qtc, suprasellar mass, hypothyroidism, hypocortisolism

## Abstract

Myxedema coma and pituitary apoplexy are well-known life-threatening endocrine emergencies. The coincidence of these entities is exceedingly rare. Myxedema coma occurring as a result of pituitary lesion is a much less seen entity. A high index of suspicion is often required for early diagnosis as it is of particular importance in improving survival outcomes. We present a rare case of a patient with myxedema coma presenting as bradycardia and hypotension secondary to pituitary apoplexy, which was confirmed on magnetic resonance imaging (MRI). The patient was managed conservatively with levothyroxine and stress doses of steroid, with the resolution of hemodynamic changes and a decrease in the size of the suprasellar mass.

## Introduction

Myxedema coma and pituitary apoplexy are well-known life-threatening endocrine emergencies. The coincidence of these entities is exceedingly rare. Myxedema coma occurring as a result of the pituitary lesion is much less seen and occurs as a result of severe hypothyroidism. The term “myxedema coma” is a misnomer as most of the patients present with an altered mental status more frequently rather than overt coma. Pituitary apoplexy generally presents with a sudden onset of excruciating headache, nausea, and visual disturbances such as diplopia, hypopituitarism, and neurological impairment [1] as a result of hemorrhage or infarction in a pituitary tumor. However, our patient had an atypical presentation with no classic apoplectic symptoms but did have biochemical evidence of hormonal deficiencies and clinical manifestations of severely reduced thyroid function.

The abstract of this case report was presented at the Southern Regional Meeting, February 2019, New Orleans, LA.

## Case presentation

**Figure 1:**
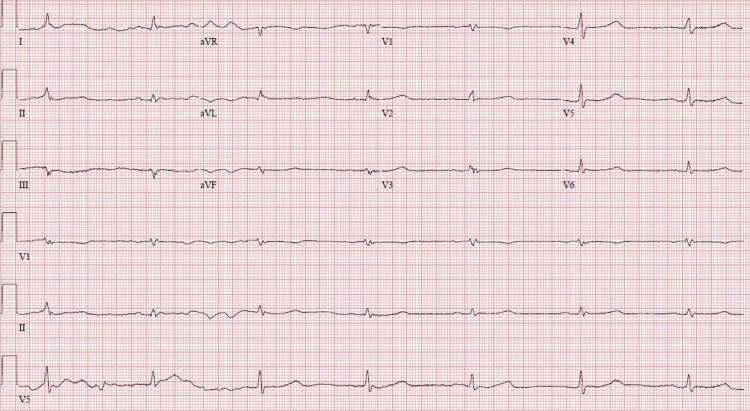
Low-voltage EKG demonstrating bradycardia, right bundle branch block, and prolonged QTc on presentation in our patient. EKG, electrocardiogram

**Figure 2:**
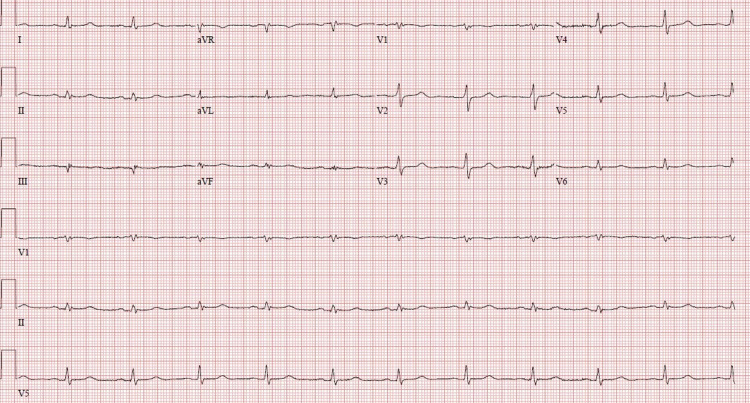
A follow-up EKG demonstrating resolution of bradycardia six hours post-treatment with levothyroxine. EKG, electrocardiogram

A 63-year-old male with a past medical history of hypertension, hyperlipidemia, and diabetes presented to our hospital with bradycardia, hypothermia, and hypotension. On presentation, his temperature was 85.4°C, blood pressure (BP) was 80/61 mm Hg, respiratory rate was 18 breaths/minute, heart rate (HR) was 40 beats minute, and saturation was 92% on 6 L nasal cannula. On physical examination, he was slow to respond, but arousable to voice, without clear orientation, and had a Glasgow coma scale of 12. An electrocardiogram (EKG) showed bradycardia, incomplete right bundle branch block, and prolonged QTc interval of 621 msec (corrected by Hodges method due to bradycardia), as shown in Figure 1. His presenting fasting blood sugar was 91 mg/dL. The chest X-ray was unremarkable. The patient was immediately started on aggressive fluid resuscitation along with a dopamine drip. Atropine was pushed intravenously with no response. Keeping a high index of suspicion for myxedema coma based on his clinical presentation, he was started on levothyroxine 200 mcg and a stress dose of hydrocortisone 100 mg every eight hours. Thyroid-stimulating hormone (TSH), free thyroxine (free T4), and cortisol level were emergently ordered. External warming with blankets was provided. Initial labs revealed TSH of 0.172 µIU/mL (reference range: 0.465-4.68 µIU/mL), free T4 of 0.41 ng/dL (reference range: 0.78-2.13 ng/dL), and cortisol of 4.5 ug/dL (reference range: 4.5 -22.7 ug/dL). Six hours after initiation of treatment, there was the resolution of bradycardia (Figure 2), prolonged QTc, and hypotension with HR of 68 beats/minute, QTc of 464 msec, and BP of 104/62 mm Hg.

**Figure 3:**
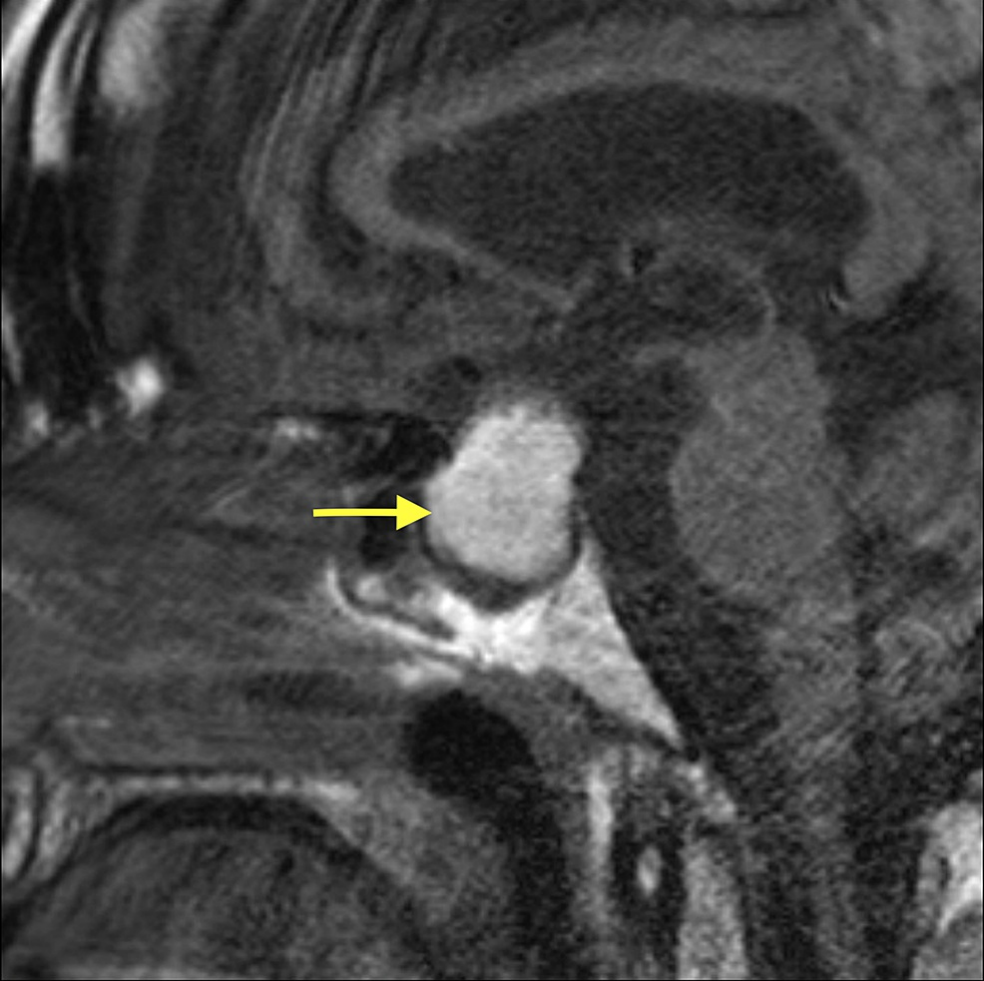
MRI demonstrating suprasellar mass (yellow arrow) measuring 2.5 x 2.2 x 2.0 cm, abutting and uplifting the optic chiasm, findings compatible with a hemorrhagic pituitary adenoma.

Considering secondary hypothyroidism and hypocortisolism, further investigations for the possible central etiology were performed. Magnetic resonance imaging (MRI) revealed suprasellar mass measuring 2.5 x 2.2 x 2.0 cm with prominent T1 hyperintensity, abutting and uplifting the optic chiasma, findings compatible with hemorrhagic pituitary microadenoma (Figure 3). Further laboratory investigations revealed decreased follicular stimulating, luteinizing, and growth hormone levels.

**Figure 4:**
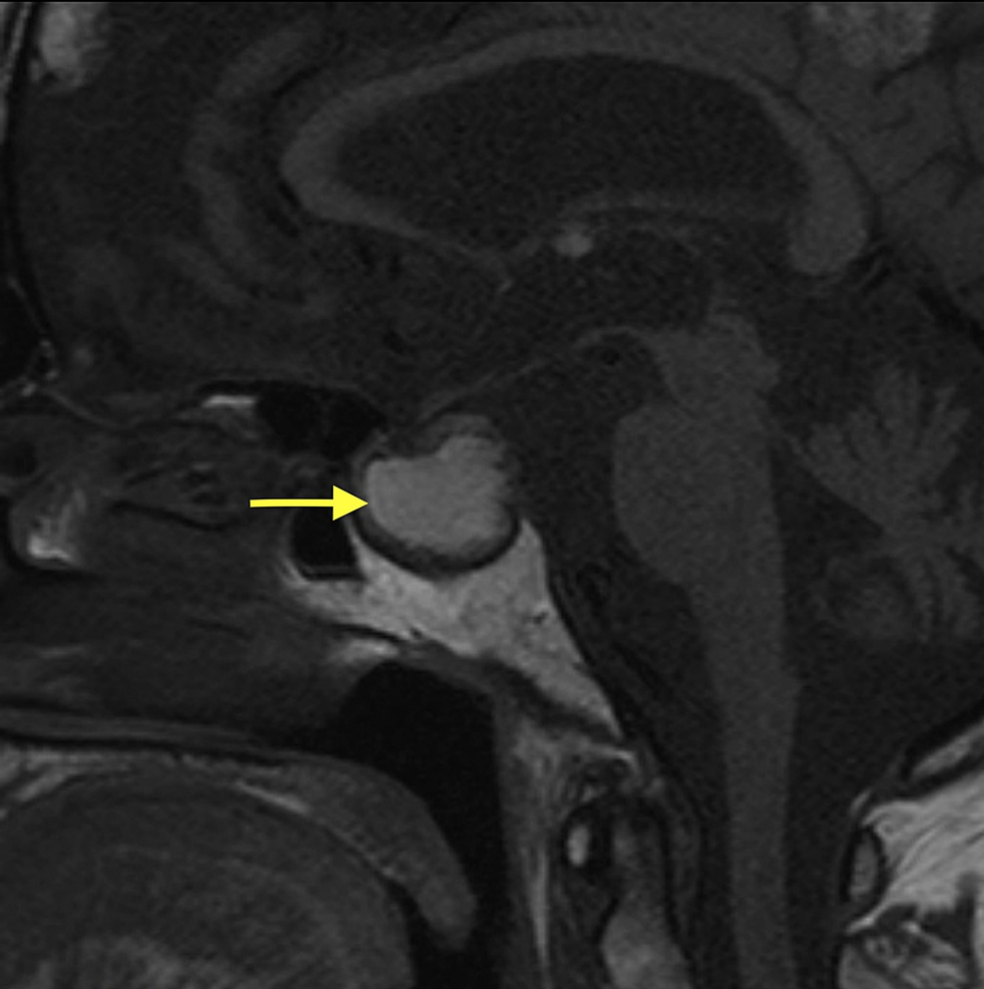
A follow-up MRI revealed reduction in the size of tumor and pituitary bleed (yellow arrow), measuring as 2.1 x 1.9 x 1.8 cm.

Once more arousable, the patient was further evaluated neurologically and did not have a visual impairment. Conservative management for pituitary apoplexy was considered with close neurological observation. Three months follow-up MRI revealed a reduction in the size of tumor and pituitary bleed, measuring as 2.1 x 1.9 x 1.8 cm (Figure 4).

## Discussion

Myxedema coma is more common in elderly females and occurs as a result of various precipitating events such as infection, gastrointestinal bleeding, cold exposure, stroke, surgery, and medications such as amiodarone or lithium [2]. The prevalence of central hypothyroidism resulting in myxedema crisis ranges from 5% to 18% [3,4]. Establishing an early diagnosis is necessary as mortality is high, ranging from 20% to 60% [5].

Thyroid hormones act intracellularly by binding to thyroid hormone response elements modifying gene transcription [6]. Effects of thyroid hormone on cardiac myocytes are mainly attributed to triiodothyronine (T3). The intracellular translocation occurs through an energy-dependent transport protein specific for T3 on the cell membrane of cardiac myocytes [7]. The signaling pathways upregulate the gene expression and protein synthesis in the myocardium. T3 regulated gene expression by phosphorylation of phospholamban is an important determinant for calcium release in the sarcoplasmic reticulum [8]. Altogether, normal thyroid function serves as an important factor for normal cardiac function.

Low serum T4 results in low intracellular T3 hormone. The decreased thyroid hormone results in diastolic hypertension, decreased blood volume, peripheral vasoconstriction, and decreased contractility of the heart, resulting in impairment of cardiovascular hemodynamics eventually leading to overt heart failure [5,9]. Consequently, severely decreased thyroid function can result in hypotension and cardiogenic shock [5], as seen in our patient. Associated EKG changes include sinus bradycardia, low voltage, ST or T wave changes, left ventricular hypertrophy, bundle branch block, and arrhythmias [4]. Prolonged QTc (as seen in our patient) along with polymorphic ventricular tachycardia has also been reported [10]. One should have a high index of suspicion in cases with altered mental status, hypothermia, and clinical feature consistent with hypothyroidism. Central hypothyroidism presents with low TSH and free T4. Once diagnosed, it is important to look for pituitary causes (secondary hypothyroidism), as seen in our patient, and/or hypothalamic causes (tertiary hypothyroidism). Also, given the atypical presentation, our patient likely had subacute or chronic hemorrhage, which gradually grew and also affected thyroid hormones level over the time.

In general, management involves protection of the airway in unconscious patients, external warming in cases of hypothermia, intravenous fluids in hypotensive patients, and antibiotics if the infection is suspected. It is important to look for an underlying precipitating cause. As gut edema may alter the absorption of oral thyroid hormone, intravenous replacement of thyroid hormone is considered the cornerstone of the treatment. Concomitant hypocortisolism is often present due to primary or secondary adrenal insufficiency, as seen in our patient. Also, thyroid replacement can cause increased cortisol clearance worsening the cortisol deficiency [5]. Therefore, it is particularly important to treat patients with stress doses of steroids along with thyroid replacement.

## Conclusions

Myxedema coma resulting from pituitary apoplexy is a rare entity. Life-threatening bradycardia and hypotension could be a part of the early syndromic presentation. Our case emphasized that early recognition of myxedema coma and its cardiac complication is important in improving survival outcomes.
